# Equivalence of Paper and Smartphone Versions of the Beck Depression Inventory-II

**DOI:** 10.3390/jcm14020500

**Published:** 2025-01-14

**Authors:** Hiroyuki Uchida, Takumi Igusa, Yurika Higashi, Minami Takeda, Kenji Tsuchiya, Senichiro Kikuchi, Kazuki Hirao

**Affiliations:** 1 Graduate School of Health Sciences, Gunma University, Maebashi 371-8514, Japan; 2 Department of Rehabilitation, Kurashiki Heisei Hospital, Kurashiki 710-0826, Japan; 3 Department of Rehabilitation, Medical Corporation Taiseikai, Uchida Hospital, Numata 378-0005, Japan; 4 Department of Occupational Therapy, Faculty of Medicine, Gunma University, Maebashi 371-8514, Japan; 5 Department of Rehabilitation, Faculty of Health Sciences, Nagano University of Health and Medicine, Nagano 381-2227, Japan

**Keywords:** patient-reported outcomes, electronic, depression, smartphone, equivalence

## Abstract

**Background:** The Beck Depression Inventory-II (BDI-II) is a widely used patient-reported outcome (PRO) tool designed to screen for depressive symptoms and assess their severity. In recent years, with advancements in digital technology, the BDI-II has been adapted for use as an electronic PRO (ePRO) tool. However, to the best of our knowledge, the reliability of the smartphone version of the BDI-II has not been thoroughly investigated. This study aimed to assess the equivalence of the traditional paper and smartphone versions of the BDI-II. **Methods:** This study employed a randomized crossover design with adults (*n* = 100) from the Gunma Prefecture, Japan. Participants completed both the paper and smartphone versions of the BDI-II at 1-week intervals. The equivalence between the two versions was evaluated using the intraclass correlation coefficient (ICC_agreement_). Additionally, Cronbach’s alpha and McDonald’s omega were calculated for both versions. **Results:** The mean age of the participants was 19.78 years (SD = 0.94, 10% male). The ICC_agreement_ between the paper and smartphone versions of the BDI-II was 0.81 (95% CI 0.74–0.87). Cronbach’s alpha was 0.88 (95% CI 0.84–0.91) for the paper version and 0.88 (95% CI 0.84–0.91) for the smartphone version. McDonald’s omega was 0.88 (95% CI 0.81–0.95) for the paper version and 0.89 (95% CI 0.85–0.93) for the smartphone version. **Conclusions:** The BDI-II is suitable for use in its smartphone version, and the smartphone version of the BDI-II is a valuable addition to the mental healthcare professional’s toolkit.

## 1. Introduction

Using patient-reported outcomes (PROs) is a key recommendation recognized globally. PROs refer to outcomes directly reported by patients about their feelings and functioning, including symptoms, physical capabilities, and quality of life, without modification or interpretation by healthcare professionals [1,2,3]. Incorporating PROs in clinical practice offers several advantages. Previous studies have demonstrated that systematically monitoring patient symptoms using PROs enhances patient–physician communication and reduces the likelihood of missed symptoms [3,4,5]. Notably, PRO-based care has been associated with improved outcomes compared to standard care [6]. Therefore, integrating PROs into routine clinical practice and research is essential. Furthermore, the recent development of digital technology and the proliferation of Internet-based interventions have increased the use of electronic PROs (ePROs), streamlining their use and expanding their application [7,8,9,10].

ePROs offer several advantages over traditional paper-version PROs. ePROs are often preferred by patients, because they may disclose more sensitive information than paper-based versions [8,10,11,12,13,14,15]. In addition, ePROs reduce score calculation errors, data entry mistakes, and missing data, thereby improving data quality and facilitating reliable analysis and reporting [8,16,17,18]. As a result, these improvements can enhance the quality of patient care and increase clinical efficiency [9,19]. In some cases, can also reduce the cost associated with data collection [9].

However, transitioning from paper-based PROs to ePROs requires careful consideration of potential challenges. In fact, the International Society for Pharmacoeconomics and Outcomes Research (ISPOR) guidelines highlight that differences in administrative methods—such as changing from circling an answer on paper to selecting it on the screen—and presentation formats, such as scrolling or font size adjustments, necessitate thorough cognitive debriefing, usability tests, and reliability evaluations (e.g., intraclass correlation coefficients [ICCs]) to ensure equivalence or superiority [8]. Many studies have been conducted on the use of ePROs across various fields [9,10,20,21]. For instance, in mental health, ePROs are increasingly utilized for screening depressive symptoms and assessing their severity [21,22,23,24,25,26]. One notable example is the Beck Depression Inventory-II (BDI-II, which has been widely investigated for its application as an ePRO [27,28,29,30].

BDI-II is one of the most widely used PROs for detecting possible depression in healthy populations and assessing the severity of depressive symptoms in diagnosed patients [31,32,33,34,35,36]. Originally developed in 1961, the BDI was slightly modified in 1979 and substantially revised in 1996 to align with contemporary diagnostic criteria for major depression [37]. The BDI-II consists of 21 items and offers notable advantages, including that it can be administered to a individuals aged 13–80 years old and completed in just 5–10 min [31,38,39]. A distinctive feature of the BDI-II is that, unlike conventional PROs, it uses descriptive response options tailored for each item, rather than using a uniform scale. This approach provides clearer context for respondents, making questions easier to understand and interpret. For instance, in assessing suicidal ideation, the response options are as follows:

0 = I do not have any thoughts of killing myself;
1 = I have thoughts of killing myself, but I would not carry them out;
2 = I would like to kill myself;
3 = I would kill myself if I had the chance.

This format enhances clarity compared to ambiguous answer choices such as “rarely” or “often” [40], allowing BDI-II to capture more nuanced details of depressive symptoms [40,41]. Because of these advantages, the BDI-II has been extensively tested for reliability and validity across diverse populations and countries. Furthermore, equivalence studies have explored its use as an ePRO [27,28,29,30,31,32,33,35,36]. However, to our knowledge, no study has specifically evaluated the equivalence of the BDI-II between its paper-based and smartphone versions.

Smartphones offer several over personal computers and tablets. With a growing number of smartphone users worldwide, most people always carry a smartphone to access the Internet [16,42]. Thus, smartphones allow more real-time access to ePRO than personal computers or tablets. Previous studies have highlighted the benefits of using smartphones as ePRO devices [16,22,24,26]. Given the advantages of smartphones, as well as the unique features of the BDI-II, which captures detailed and nuanced depressive symptoms, a smartphone version of the BDI-II could assess depressive symptoms in real time [40,41]. In turn, this may reduce the number of depressive symptoms overlooked by physicians and improve communication between patients and healthcare providers [3,4,5]. For this reason, it is important to develop a smartphone version of the BDI-II.

However, the ISPOR guidelines emphasize that differences in question phrasing and presentation formats between ePROs and original paper-based versions could adversely affect their reliability and validity [8]. Moreover, the type of device used may also influence these factors [8]. Therefore, a comparison of the ePRO and the original PRO should be conducted on a device-by-device basis.

This study aimed to evaluate the equivalence of the paper-based and smartphone-based versions of the BDI-II in accordance with the ISPOR guidelines. The hypothesis of this study was that the scores obtained from the smartphone version of the BDI-II would be equivalent to those from the paper-based version.

## 2. Material and Methods

### 2.1. Study Design and Ethics

**Figure 1:**
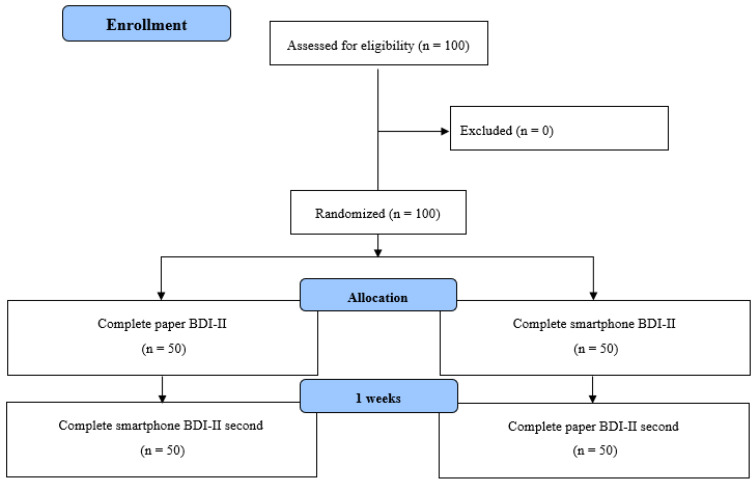
Flowchart of the study.

This randomized crossover study aimed to evaluate the format-to-format equivalence of the paper-based and smartphone versions of the BDI-II (Figure 1). The study participants were recruited from the Gunma University in Gunma, Japan, from October 2023 to December 2023. The study was conducted according to the ISPOR guidelines and was approved by the Ethical Review Board for Medical Research Involving Human Subjects of Gunma University (approval no. HS2022-109) [8]. Written informed consent was obtained from each participant before study participation.

### 2.2. Participants and Procedure

Participants in this study were recruited from Gunma University in the Gunma Prefecture, Japan, using advertisements, emails, and social networking services. The eligibility criteria for this study were as follows: (1) 18 years of age or older, (2) male or female, (3) smartphone owner, (4) native Japanese speaker, and (5) obtained informed consent. Eligible participants were randomly assigned to either the smartphone-first (groups responding to the smartphone-based version BDI-II first) or paper-first (groups responding to the paper-based version BDI-II first) group after providing their demographic information (age and gender) and lifestyle habits (drinking, exercise, and smoking habits). One week after the first survey response, the smartphone-first group responded to the paper-based version of the BDI-II and the paper-first group responded to the smartphone version of the BDI-II. This 1-week interval was chosen based on a prior study investigating the equivalence of paper-based and electronic PROs, aimed at reducing potential recall and carryover effects [26]. These questionnaires were administered under the researcher’s supervision and conducted in a controlled environment, minimizing the risk of omitted responses and standardizing test conditions by reducing noise, distraction, and fatigue.

### 2.3. Randomization

Participants were randomly assigned to the paper- or smartphone-first groups in a 1:1 ratio before completing the survey. The randomized list was generated using the permuted block method (block size 4) and Excel software by a third-party unrelated to the study and sent to the central registry center of Kurashiki Heisei Hospital in Okayama, Japan. Once the participants were enrolled in the study, the researchers informed the central registry center at Kurashiki Heisei Hospital, which performed the random assignment.

### 2.4. Sample Size

The sample size was determined on the basis of the Consensus-based Standards for the Selection of Health Measurement Instruments (COSMIN) initiative and the ISPOR guidelines. The ISPOR guidelines used Walter’s methodology to calculate the sample size required for ICCs. With an ICC of 0.85 in the underlying population and two assessments conducted at 80% power, 43 complete data points would be needed to conform that the ICC of the true population exceeds 0.70 with 95% confidence [8,43]. However, the COSMIN initiative recommends a sample size of at least 100 to achieve adequate statistical power for assessing testing–retesting reliability (i.e., equivalence) [44]. Thus, the target sample size for this study was set at 100.

### 2.5. Measure: Beck Depression Inventory-II

BDI-II is a 21-item self-administered questionnaire designed to measure the severity of depressive symptoms experienced over the previous two weeks [31,38]. Each item is rated using a 4-point Likert scale ranging from 0 to 3 points. The total scores ranged from 0 to 63 points, with higher scores indicating greater severity of depressive symptoms. Previous studies have well documented the reliability and validity of BDI-II [31,32,33,35,37,39,40,45,46,47,48].

### 2.6. Software

An electronic version of the BDI-II was provided to participants via Google Forms on their smartphones. The question items, answer choices, and question order in the smartphone version of the BDI-II were identical to those in the paper version. All questions for each survey were displayed on the screen, allowing participants to scroll down to answer subsequent questions. Responses were selected by tapping radio buttons on the screen. It was impossible to skip questions or select multiple answers for each item, though participants could revise their previous answers.

### 2.7. Statistical Analysis

The ISPOR guidelines categorize modifications from PRO to ePRO into three levels, namely Minor, Moderate, and Substantial [8]. Minor modifications involve transferring a scale from paper to screen without significantly altering font size, item content, recall period, or response options. Moderate modifications include splitting items into multiple screens, significantly reducing font size, and requiring patients to scroll to view all text or responses. Substantial modifications include deleting items or drastically changing item text.

In this study, the font size of the smartphone version of the BDI-II administered was different from that of the paper version and scrolling was required to navigate between questions. These changes correspond to moderate modifications under the ISPOR guidelines. When PROs undergo such modifications, it is recommended to formally establish the equivalence of electronic measures [8]. Therefore, we adopted a randomized crossover design to evaluate the equivalence of the smartphone version of the BDI-II and the paper version of the BDI-II and calculated the ICC_agreement_ and its 95% confidence interval based on a two-way random-effects model. In this study, we adopted a randomized crossover design for several reasons and used ICC_agreement_ as the measure of equivalence. ICC is one of the most commonly used measures for assessing equivalence between ePROs and PROs, and the ISPOR guidelines recommend using a crossover design in such cases [8,13,24,49,50,51]. Another advantage of using crossover designs is that they improve the power of the test, making it possible to evaluate equivalence with fewer samples [8]. In addition, ICC_consistency_, the Pearson correlation coefficient, and the Spearman correlation coefficient only take random error into account, as opposed to systematic errors; however, ICC_agreement_ considers both random and systematic errors, and it has been suggested that it is an extremely rigorous parameter for evaluating equivalence [8,44,52]. The ICC is expressed as a value between 0 and 1, with an ICC of 0.70 or greater indicating good reliability [44,52]. We also calculated Cronbach’s alpha and McDonald’s omega and their 95% CIs to assess the internal consistency of paper PRO and ePRO. Cronbach’s alpha and McDonald’s omega are expressed as 0–1, with values above 0.7 indicating good internal consistency [52,53].

In addition, linear mixed models (LMMs) were used to evaluate carryover effects [54]. A LMM considers the administration format (paper or smartphone), the order of administration, and their interaction as fixed effects, while treating participants as a random effect. Statistical significance was set at *p* < 0.05 with two-tailed tests. All analyses were performed using R version 4.3.1. We used the lmerTest package and the lme4 package to conduct the LMM analysis. In addition, we used the irr package to calculate ICC_agreement_, and the psych and MBESS packages for Cronbach’s alpha and McDonald’s omega.

## 3. Results

### 3.1. Characteristics of the Study Participants

Of the 100 participants who met the eligibility criteria, all completed both the paper- and smartphone-based versions of the BDI-II and provided complete data. Table 1 presents the demographic and lifestyle characteristics of the participants. The mean age was 19.78 years (SD = 0.94), with ten participants (10%) being male. Seven participants (7%) reported a drinking habit, none (0%) had a smoking habit, and 28 participants (28%) engaged in regular exercise.

### 3.2. Mean and LMM Results for the Paper- and Smartphone-First Groups

Table 2 presents the means and LMM results for the paper- and smartphone-first groups. In the paper-first group, the mean BDI-II score for the paper version was 6.82 (SD = 5.01), while in the smartphone-first group, the mean score was 7.78 (SD = 7.24). For the smartphone version, the mean BDI-II score was 5.82 (SD = 4.88) in the paper-first group, and 9.56 (SD = 7.36) in the smartphone-first group. The interaction between administration format and order was not significant in the LMM (*p* = 0.29; 95% CI −2.20–0.64), indicating that no carryover effect was observed.

### 3.3. Agreement Between the Smartphone and Paper Versions of BDI-II

Table 3 shows the results of the ICC_agreement_ for the BDI-II scores. The ICC_agreement_ between the paper-based and smartphone-based BDI-II scores was 0.81 (95% CI 0.74–0.87).

### 3.4. Internal Consistency (Cronbach’s Alpha and McDonald’s Omega) for the Two Groups

Table 4 shows the Cronbach’s alpha and McDonald’s omega values for the BDI-II in each group. The Cronbach’s alpha for the paper version was 0.88 (95% CI 0.84–0.91) and 0.88 (95% CI 0.84–0.91) for the smartphone version. McDonald’s omega for the paper version was 0.88 (95% CI 0.81–0.95) and 0.89 (95% CI 0.85–0.93) for the smartphone version.

## 4. Discussion

In addition to the established advantages of PROs, such as reducing missed symptoms and improving communication between patients and healthcare providers, the use of ePROs is increasing worldwide, driven by advancements in digital technology and the internet [3,4,5,6,7,8,9,10]. ePROs are widely used in the field of mental health, particularly for screening depressive symptoms and assessing their severity, with the BDI-II being one such tool [21,22,23,24,25,26,27,28,29,30,31,32,33,35,36]. However, to the best of our knowledge, no studies have evaluated the equivalence between the paper-based and smartphone-based versions of the BDI-II. Given the global rise in smartphone users, the ubiquity of smartphones, and the increasing number of people using them to access the internet, the smartphone version of the BDI-II presents a promising alternative to PCs and other devices [16,42]. Therefore, this study aimed to evaluate the equivalence between the smartphone-based and paper-based versions of the BDI-II, following the ISPOR guidelines.

The ICC_agreement_ for the BDI-II in this study was 0.81 (95% CI 0.74–0.87). Cronbach’s alpha was 0.88 (95% CI 0.84–0.91) for both the smartphone and paper versions, indicating comparable reliability. The internal consistency measured by McDonald’s omega was 0.89 (95% CI 0.85–0.93) for the smartphone version and 0.88 (95% CI 0.81 to 0.95) for the paper version, suggesting almost identical internal consistency. Previous studies indicate that an ICC, Cronbach’s alpha, and McDonald’s omega value of 0.70 or higher is considered good [44,52,53]. In addition, the 95% CI results for ICC_agreement_, Cronbach’s alpha, and McDonald’s omega for the BDI-II in this study suggested a 95% probability that even in the worst-case scenario, these values would remain at 0.7 or higher [55]. Thus, the smartphone version of the BDI-II is likely to yield results similar to those of the paper version. Given the study results and the convenience of smartphones, using the BDI-II via smartphones may be a more promising strategy than using other devices, such as PCs or tablets. However, ICC and Cronbach’s alpha should be at least 0.7 for population-level use and between 0.85 and 0.95 for individual-level use [8]. Therefore, while the smartphone version of the BDI-II is suitable for population-level use, it may not be appropriate for individual-level use.

This study provided evidence that the smartphone version of the BDI-II is equivalent to the paper version. However, despite these promising results, the conventional paper-based version may still be more appropriate for use with older populations. Previous studies suggested that older adults’ e-health literacy—defined as the ability to effectively use digital technologies with internet access, such as smartphones, wearables, tablets, and computers in order to make health decisions—should be considered when introducing digital technologies [56,57,58,59]. Additionally, the utilization rate of e-health services tends to be low in older populations [56,57,59]. A previous study investigating the equivalence of smartphone-based ePROs also indicated that older adults may have been hesitant to participate, either due to limited experience with smartphones or not owing one [24]. Similarly to these findings, it cannot be denied that older individuals may experience resistance to using the smartphone version of the BDI-II. As such, the traditional paper version may still be necessary when administering the BDI-II to older populations. For this reason, it is expected that in both clinical practice and research, the smartphone version and paper version of the BDI-II should be used appropriately, depending on the patient’s age.

Our findings contribute to expanding the evidence for using the BDI-II as an ePRO and provide valuable insights for future equivalence studies. To our knowledge, no studies have specifically evaluated whether the BDI-II could be used as an ePRO on smartphones alone, although several studies have explored its use on other devices or across multiple platforms. For instance, the equivalence of the internet and paper versions of the BDI-II was assessed in 87 patients from primary and psychiatric care in Sweden, yielding a Pearson correlation coefficient of 0.89 between the two versions [27]. The Cronbach’s alpha for the Internet version ranged from 0.87 to 0.89, while the paper version’s Cronbach’s alpha was between 0.89 and 0.90 [27]. In addition, the equivalence between the computer version of the BDI-II and the paper version was tested in 180 college students, with a correlation coefficient of 0.98 [28]. The Cronbach’s alphas for the computer and paper versions of the BDI-II were 0.91 and 0.88, respectively [28]. Another study investigating the equivalence of the internet and paper versions of the BDI-II in 494 Swedish patients with panic disorder found a Pearson correlation coefficient of 0.94, with Cronbach’s alphas for the internet and paper versions ranging from 0.88 to 0.92 and 0.89–0.90, respectively [29]. A study validating the web-based version of the BDI-II in 185 Spanish university staff also found a Cronbach’s alpha of 0.90 [30]. However, several methodological problems in these studies limit the generalizability of the BDI-II as an ePRO. While many studies use the Pearson or Spearman correlation coefficients to investigate equivalence, their measurements are not considered highly rigorous for equivalence evaluation because they do not account for systematic errors [44,52]. Therefore, assuming equivalence between the paper and computer or web versions of the BDI-II based solely on these correlations may be misleading. In addition, for internal consistency, many studies rely on Cronbach’s alpha, which is a widely used measure, but assumes that the tau equivalence model is met. This assumption may not hold for many PRO measures, potentially leading to the over- or underestimation of internal consistency [53]. To address these statistical concerns, we used the ICC_agreement_ which accounts for both systematic and random errors. In addition, we calculated McDonald’s omega, a more accurate measure of internal consistency than Cronbach’s alpha. Consequently, our study provides a more precise validation of BDI-II equivalence and internal consistency, addressing issues that previous studies encountered when adapting the BDI-II to ePRO formats.

Nevertheless, several limitations exist in this study. First, the participants represented a relatively young Japanese population (ages 18–22). Therefore, the results may not be applicable to other countries or age groups. Second, this study did not assess differential item functioning (DIF). The ISPOR guidelines suggest performing item response theory (IRT) analysis to evaluate DIF in the scale [8]. However, conducting a DIF analysis for the BDI-II in this study was challenging because IRT requires a sample of at least 200 individuals [8]. Future studies should evaluate DIF in the smartphone version of BDI-II with a larger sample size. Third, while the crossover design was used, potential carryover cannot be entirely ruled out. Thus, our results should be interpreted with caution. However, based on previous research, a one-week interval between the first and second survey was chosen to minimize carryover effects, and no statistically significant difference in carryover effects was observed. Fourth, minor modifications recommended by the ISPOR guidelines, such as cognitive debriefing and usability testing, were not performed to minimize participant burden [8]. As a result, the usability of the smartphone version of the BDI-II remains unclear. Additionally, previous studies have suggested that healthcare providers may have negative attitudes toward implementing digital technologies in clinical practice [60]. Future research should include cognitive debriefing and usability testing for both the recipients and the providers of the smartphone version of the BDI-II. Fifth, this study was conducted with a sample of 100 people extracted from a single facility. Although the sample size was consistent with the COSMIN recommendations, it remains relatively small [44]. Moreover, participants were recruited from only one facility, which limits the generalizability of our results. Therefore, future research should involve larger sample sizes drawn from multiple facilities to replicate these findings.

## 5. Conclusions

This study demonstrated the equivalence between the smartphone and paper versions of the BDI-II, indicating that the BDI-II could be effectively used in clinical and research settings through smartphones, which are convenient and user-friendly tools. With the continued growth of digital technology in mental health, the smartphone version of the BDI-II will be an important asset for mental healthcare professionals. However, the traditional paper versions of the BDI-II may still be recommended for older individuals. Future studies should aim to recruit large, diverse samples from multiple institutions, and replicate the findings of the present study.

## Figures and Tables

**Table 1:** Baseline characteristics of the two groups.

	Total (*n* = 100)	Paper-First (*n* = 50)	Smartphone-First (*n* = 50)
Characteristics			
Age (years)	19.78 (0.94)	19.78 (0.86)	19.78 (1.02)
Sex			
Male	10 (10%)	5 (10%)	5 (10%)
Female	90 (90%)	45 (90%)	45 (90%)
Drinker			
Yes	7 (7%)	3 (6%)	4 (8%)
No	93 (93%)	47 (94%)	46 (92%)
Smoker			
Yes	0 (0%)	0 (0%)	0 (0%)
No	100 (100%)	50 (100%)	50 (100%)
Exercise habits			
Presence	28 (28%)	17 (34%)	11 (22%)
Absence	72 (72%)	33 (66%)	39 (78%)

Data are presented as means (standard deviation) or numbers (%).

**Table 2:** Mean and LMM results for the paper-first and smartphone-first groups.

	Total (*n* = 100)	Paper-First (*n* = 50)	Smartphone-First (*n* = 50)	LMM			
Outcomes	Mean (SD)	Mean (SD)	Mean (SD)	Effect	Estimate	*p*	95% CI
BDI-II							
Paper	7.30 (6.21)	6.82 (5.01)	7.78 (7.24)	Format	−1	0.06	−2.01–0.01
Smartphone	7.69 (6.49)	5.82 (4.88)	9.56 (7.36)	Order	2.74	0.03	0.30–5.18
				Interaction	−0.78	0.29	−2.20–0.64
BDI-II: Beck Depression Inventory-II							
SD: Standard deviation							
LMM: Linear mixed models							
CI: Confidence interval							

**Table 3:** Agreement between the smartphone and paper versions of the BDI-II.

Outcomes	ICC_agreement_	95% CI
BDI-II	0.81	0.74–0.87
BDI-II: Beck Depression Inventory-II		
ICC: Intraclass correlation coefficient		
CI: Confidence interval		

**Table 4:** Internal consistency (Cronbach’s alpha and McDonald’s omega) for the two groups.

Outcomes	Cronbach’s Alpha	95% CI	McDonald’s Omega	95% CI
BDI-II				
Paper	0.88	0.84–0.91	0.88	0.81–0.95
Smartphone	0.88	0.84–0.91	0.89	0.85–0.93

BDI-II: Beck Depression Inventory-II; CI: confidence interval.

## Data Availability

Due to the restrictions imposed by the Ethics Committee, the data used in this study cannot be publicly shared. However, data can be obtained from the corresponding author upon reasonable request.
